# Src as the link between inflammation and cancer

**DOI:** 10.3389/fphys.2013.00416

**Published:** 2014-01-16

**Authors:** Sandy T. Liu, Hung Pham, Stephen J. Pandol, Andrzej Ptasznik

**Affiliations:** ^1^Department of Medicine, Cedars-Sinai Medical CenterLos Angeles, CA, USA; ^2^Department of Medicine, David Geffen School of Medicine, University of California-Los AngelesCA, USA; ^3^Department of Medicine, Veterans AffairsLos Angeles, CA, USA

**Keywords:** inflammation, cancer, Src, cytokines, chronic pancreatitis, pancreatic cancer

## Abstract

Although a causal link between chronic inflammation and cancer has been established, the exact molecular mechanism linking inflammation to cancer remains largely unknown. It was previously postulated that molecular switches responsible for cancer cell development, and for infiltration of inflammatory cells into cancer, were divided into a distinct set of intracellular proteins and signaling pathways. However, recent evidence suggests that both tumor cells and tumor-infiltrating immune cells utilize the same kinases, mostly that of Src family, to facilitate cancer development and progression. In the past few years several groups have found that Src activation both in cancer and inflammatory cells is mainly driven by pro-inflammatory cytokines within the tumor microenvironment. Here we evaluate the cross talks between Src kinase pathways in immune cells and cancer cells. We conclude that Src might serve as a critical mechanistic link between inflammation and cancer, mediating and propagating a cycle between immune and tissue cells that can ultimately lead to the development and progression of cancer.

## Introduction

Inflammation is a vital defensive response that serve critical roles in a variety of physiological situations, and when dysregulated, can contribute to the pathogenesis of many diseases. Chronic inflammation is a well-documented risk for promoting cancer (Coussens and Werb, 2002; Balkwill et al., 2005; Mantovani et al., 2008), particularly in the pancreas and GI tract (Guerra et al., 2007; Terzić et al., 2010). Chronic pancreatitis is long-standing inflammation of the pancreas associated with an increased risk (~20-fold) for pancreatic cancer. This projects a serious clinical problem as pancreatic cancer is a highly lethal disease with the worst prognosis of all the major malignancies; for all stages combined, and a 5-year survival rate of 5% (Yadav et al., 2011). Similarly, uncontrolled inflammatory bowel disease poses a significant risk factor for colorectal cancer. When compared to the general population matched for age, sex, and years at risk, there is a 18-fold increase in Crohn's disease and a 19-fold increase in ulcerative colitis, (Bernstein et al., 2001; Eaden et al., 2001; Itzkowitz and Yio, 2004; Ullman and Itzkowitz, 2011). Interestingly, many environmental cancer risk factors, including alcohol overuse, smoking, chronic infections and obesity, can trigger some form of chronic inflammation, largely in the pancreas and colon (Trinchieri, 2012). These environmental risk factors seemingly facilitate the development and progression of cancer mostly through the induction of chronic persistent inflammation in these tissues.

Although many studies point to an association between inflammation and cancer, the mechanistic signaling basis of this linkage is not well understood. The importance of Src family kinases in colon and pancreatic cancer development is known for many years and is well established (Staley et al., 1997; Lutz et al., 1998; Aligayer et al., 2002). Recent evidence has shown that Src signaling network is also very important in movement and infiltration of immune cells into tumor (Balkwill, 2004; Kulbe et al., 2004). Several groups have found that Src activation in cancer and immune inflammatory cells are mediated by inflammatory cytokines within the tumor microenvironment. Given that Src is overactive in both tumor cells and in tumor-infiltrating immune cells, and is also involved in cytokine-mediated cross talk between cancer and inflammatory cells—Src may be a critical link between inflammation and cancer. We illustrate and expound on this concept using the model of chronic pancreatitis and pancreatic cancer.

## Persistent inflammation increases cancer risk in pancreas

Chronic pancreatitis highlights an important role for chronic inflammation in the development of cancer. Chronic pancreatitis is the most consistent risk factor for pancreatic cancer and alone increases the risk of developing pancreatic cancer by 10–20-fold (Dítě et al., 2012). Many of the environmental cancer risk factors can initially induce chronic inflammation that subsequently leads to pancreatic cancer. Recurrent pancreatic injury from alcohol abuse, smoking, high-fat diet, diabetes, and genetic predisposition, induces a pro-inflammatory environment consisting of various types of immune cells, cytokines, chemokines, and growth factors that, when dysregulated and persistent, can ultimately lead to the development and progression of cancer (Lowenfels et al., 2001; Shoelson et al., 2007; Pannala et al., 2009; Momi et al., 2012).

Alcohol abuse is a major cause of acute and chronic pancreatitis. The disease usually presents as an acute episode of pancreatitis and progress with additional exacerbations that can lead to chronic pancreatitis, characterized by a sequence of necrotic and fibrotic events. The initial tissue injures are associated with cytokine release during necro-inflammation and appears to include premature intracellular activation of digestive enzymes, leading to autodigestion. Alcohol metabolism causes release of endogenous hydrolases from pancreatic lysozymes, which are responsible for premature activation of trypsinogen leading to intrapancreatic autodigestion and inflammation (Talamini et al., 1999). Reactive oxygen species generated results in further pancreatic tissue injury, and further release of pro-inflammatory cytokines and chemokines (Shi et al., 2005).

In addition, alcohol when combined with cigarette smoking exacerbates the chronic inflammatory process (Go et al., 2005; Maisonneuve et al., 2005; Wiśniewska et al., 2013). Cigarette smoking contributes to the development of chronic pancreatitis by inducing cytokine release and inflammation. Smoking is the major risk factor for the development of pancreatic cancer accounting for 20–30% of cases (Lowenfels et al., 2001). In experimental models, nicotine stimulated an acute inflammatory reaction in the pancreas, which progressed to chronic pancreatitis after repeated sessions of smoking-induced acute pancreatic inflammation. These nicotine-induced inflammatory events are clearly associated with the release of pro-inflammatory cytokines (Nordskog et al., 2003).

Both central and overall obesity are associated with increased risk for pancreatic cancer (Pannala et al., 2009). Although the exact mechanism of obesity to pancreatic cancer is unclear, the major issues revolve around chronic inflammation, glucose intolerance, hyperinsulinemia, insulin resistance, and oxidative stress. Inflammation, along with the immune system plays a vital role in the development of insulin resistance, diabetes, and ultimately pancreatic cancer. Adipose tissue is involved in the release of cytokines and chemokines including TNF-α, IL-6, MCP-1, CXCL12, CCL5, CCL20, that lead to the recruitment of pro-inflammatory cells into adipose tissue (Shoelson et al., 2007; Sell et al., 2012). Obese individuals also exhibit lower circulating levels of anti-inflammatory adipokines that sustains a low-grade systemic inflammation.

Hereditary pancreatitis is a rare autosomal dominant condition caused by gain-of-function mutations in the cationic trypsinogen gene (PRSS1) and is responsible for <1% of all forms of pancreatitis. Mutant PRSS1 gene causes premature activation or impairs the deactivation of trypsin leading to recurrent injury, cytokine release, and inflammation. The risk of developing pancreatic cancer is 53 times higher when compared to the risk in unaffected individuals. Of the patients who progress to chronic pancreatitis, the risk of developing pancreatic cancer by age 70 years is approximately 40%. Pancreatic inflammation also occurs at a much younger age in this group of patients. In addition, Lowenfels et al. reported a 2-fold increased risk of developing pancreatic cancer in smokers with hereditary pancreatitis as compared to non-smokers. Pancreatic cancer also developed 20 years earlier in smokers than in non-smokers (Howes et al., 2004; Rebours et al., 2009), suggesting that nicotine-induced release of cytokines and inflammation can rapidly accelerate the promotion and development of cancer in these patients.

## Inflammatory cells infiltrate tumor in pancreas

**Figure 1:**
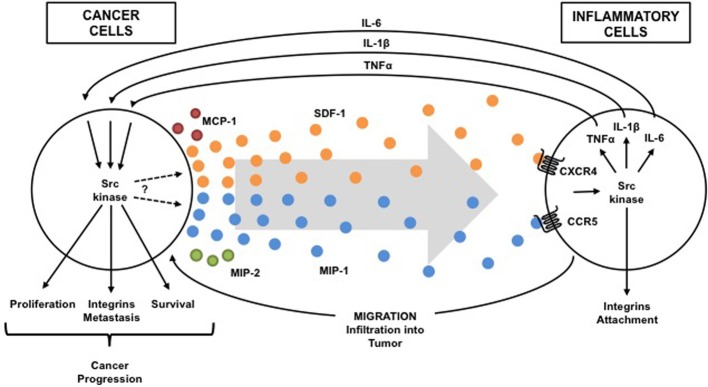
**Src-dependent cross talk between inflammatory and cancer cells**. Src activation is driven in both inflammatory cells and cancer cells by pro-inflammatory cytokines within the tumor microenvironment. Activation of Src kinases in immune cells by tumor-secreted cytokines (chemokines: SDF-1, MIP-1, MCP-1, MIP-2, etc.) induces production of cytokines (TNF-a, IL-1b, IL-6, etc.) that reciprocally activate Src in cancer cells to promote cancer progression and to induce more cytokine (chemokine) that attract more inflammatory cells into the tumor. Thus, a Src-dependent condition exists in which one problem causes another problem that makes the first problem worse (vicious cycle).

Since the role of various immune cells (including lymphocytes, granulocytes, and macrophages) in pancreatic inflammation and cancer has been discussed elsewhere (Mantovani et al., 2008), this review will focus on studies of macrophages as Src kinase-dependent and cytokine-mediated linkage between inflammation and cancer seems most apparent in these cells. Tumor-associated macrophages are key players in pancreatic inflammation and cancer and an important source of cytokines (Feig et al., 2012; Liou et al., 2013). As described above, chronic pancreatitis is often initiated by environmental risk factors, leads to permanent damage of pancreas, and is a consistent risk factor for pancreatic cancer. Chronic pancreatitis is characterized by marked stroma formation with a high number of infiltrating macrophages and myofiroblastic-like stellate cells, which are believed to play a central role in initiating inflammation and disease progression (Erkan et al., 2012). In response to pancreatic injury (alcohol abuse, cigarette smoking, obesity, mutations in genetically predisposed persons, etc.), inflammatory signals and chemokines production are upregulated leading to infiltration of leukocytes and stellate cells to the damaged acinar cells. Inflammatory cells that are recruited in turn secrete several cytokines, including chemokines, interleukins, and interferons, that contribute to cancer growth, invasion, and metastasis (Figure 1, Table 1).

**Table 1:** **Src tyrosine kinase family and cytokine/chemokine interaction in immune and cancer cells**.

**Src TYROSINE KINASE FAMILY MEDIATE CYTOKINE/CHEMOKINE PRODUCTION**			
**Src kinase family**	**Cytokines/chemokines**	**Source**	**References**
Src	MIP-1α, MCP-1, MIP-2	Acinar cells	Ramnath et al., 2009
Lyn	TNF-α	Macrophages	Tomkowicz et al., 2006
Hck	TNF-α, IL-6	Colorectal cancer	Smolinska et al., 2011
Src	TNF-α, IL-6	Macrophages	Sarang et al., 2011
Lyn	IL-1β	Macrophages	Cheung et al., 2008
**CYTOKINES ACTIVATE Src TYROSINE KINASE FAMILY**			
**Cytokines**	**Src kinase family**	**Source**	
TNF-α	Src	Acinar cells	Satoh et al., 2005
IL-6	Src	Gastric cancer cells	Lin et al., 2007
IL-6	Hck	Myeloma cells	Podar et al., 2004
IL-6	Fyn, Lyn Hck	Myeloma cells	Hallek et al., 1997
**CHEMOKINES ACTIVATE Src TYROSINE KINASE FAMILY**			
**Chemokines**	**Src kinase family**	**Source**	
SDF-1	Src	Ductal cells	Kayali et al., 2003
SDF-1	Lyn	Macrophages	Malik et al., 2008
SDF-1	Lyn	B lymphocytes	Nakata et al., 2006
SDF-1	Lck	T lymphocytes	Inngjerdingen et al., 2002
MIP-1-β	Lyn	Macrophages	Tomkowicz et al., 2006
RANTES	Lyn	Macrophages	Cheung et al., 2009

Numerous experimental studies have suggested an important role of macrophages in generating the microenvironment for both chronic pancreatitis and tumor cells, thus highlighting a similarity between stroma composition in chronic pancreatitis and pancreatic cancer. Macrophages are derived from circulating peripheral monocytes mostly in response to chemokine monocyte chemoattractant protein 1 (MCP-1). Several other chemokines, including MIP-1, MIP-2, and SDF-1, are also increased at the site of inflammation attracting leukocytes and tissue precursors to the injured pancreas (Spaeth et al., 2008). In turn, macrophages, other leukocytes, and stellate cells, which all infiltrate the tumor, release cytokines, including IL-1, IL-6 and TNF that directly effect cancer cell proliferation, and movement/attachment. This process promotes cancer development and progression (Figure 1, Table 1). It is also possible that the cytokine-mediated persistent activation of certain key intracellular signaling pathways, which occurs during chronic inflammation, might inhibit apoptosis and prevent the elimination of genetically altered, precancerous and cancerous cells.

## Src activation contributes to both inflammation and cancer in pancreas

Src was the first transforming protein discovered and isolated (Rous, 1911; Stehelin et al., 1976; Brugge and Erikson, 1997) and was also the first gene product with protein tyrosine kinase activity (Hunter and Sefton, 1980). The Src family kinases comprise of nine non-receptor protein tyrosine kinases that share similar structure and function. Src family kinases have a critical role in cell adhesion, proliferation, survival, and invasion, including cell movement, and activation of cytokine receptors. Numerous groups have found that hyper-activation and/or overexpression of Src family kinases are critical to various types of cancers.

Expression of several members of the Src-family tyrosine kinases, including Src, Fyn, Yes, Fgr and Lyn has been demonstrated in pancreatic cancer cell lines and primary cells. The expression of Lyn kinase is the most abundant in these cells (Fu et al., 2006). Numerous studies have shown that elevated Src-family kinase activity in human pancreatic carcinomas (when compared to normal pancreatic cells) not only contributes to pancreatic cancer growth, but also to invasion and metastasis (Lutz et al., 1998; Trevino et al., 2006; Yokoi et al., 2011). Src kinases and oncogenic Ras, PI3K, p38MAPK and Dynamin-2 have been shown to co-operatively stimulate the growth, metastatic migration and invasion of pancreatic carcinoma (Summy et al., 2005; Shields et al., 2011).

Src activation has been observed in circulating blood monocytes and tissue macrophages in chronic pancreatitis, as well as in tumor-associated macrophages and acinar cells in pancreatic cancer (Yokoi et al., 2011). Elevated level of activity of Src in inflammatory monocytes/macrophages was proposed as a biomarker for pancreatic cancer (Coppola, 2000; Yokoi et al., 2011). However, no oncogenic mutations responsible for Src activation in inflammatory and cancer cells in the pancreas have yet been identified. Thus, Src activation is likely a result of underlying inflammation and the consequence of a cytokine-mediated inflammatory microenvironment during malignant transformation and progression. It seems that the signal activating Src kinases is within the inflammatory microenvironment without the necessity of the Src mutation. Consequently, several groups have found that Src activation is driven by pro-inflammatory cytokines, and inversely, the cytokine production is driven by Src kinases, in various types of cancer and inflammatory cells, as summarized in Table 1.

As previously discussed, in response to pancreatic injury, chemokine production is upregulated leading to infiltration of leukocytes and stellate cells to the injured acinar cells. Rather limited information is available on the exact role of Src kinases in chemokine production in pancreatic inflammation and cancer (note the question mark in the Figure 1). However, Src kinases involvement in the secretion of several chemokines was demonstrated in pancreatic acinar cells (Ramnath et al., 2009) and ductal cells (Ungefroren et al., 2011). The pretreatment of pancreatic acini with Src kinase inhibitors markedly decreased MCP-1, MIP-1, and MIP-2 production after stimulation with the substance-P (Ramnath et al., 2009). Substance-P is known to play a role in pathogenesis of cerulein-induced pancreatitis and pancreatic cancer invasion (Ramnath and Bhatia, 2006; Ito et al., 2007).

Accordingly, it also has been shown that the expression of CCR5 receptor for MIP-1, MCP-2 and RANTES, is upregulated in chronic pancreatitis in human tissue, as compared with the healthy pancreas, and the majority of CCR5-positive cells were infiltrating macrophages (Goecke et al., 2000). Similarly, the expression of the CCR5 chemokine receptor and its ligands (MIP-1, MCP-2, RANTES) was significantly increased in the mouse pancreas during cerulein-induced pancreatitis (Goecke et al., 2000; Duell et al., 2006). On the other hand, the SDF-1 chemokine signaling in pancreas and in the other tissues is also dependent on Src family kinases (Takatomo et al., 2000; Nakata et al., 2006; Malik et al., 2008). Src family kinases are downstream intracellular targets of CXCR4 receptor, and are required for the SDF-1—mediated cell movement and attachment (Nakata et al., 2006; Malik et al., 2008). The SDF-1-CXCR4 ligand receptor axis induces pancreatic cancer cell invasion, and the Src-mediated SDF-1 signaling is also an obligatory component of pancreatic regeneration (Takatomo et al., 2000; Kayali et al., 2003; Gao et al., 2010).

In addition, we have previously shown the SDF-1-CXCR4-Src signaling axis is crucial for the movement and invasiveness of inflammatory leukocytes, in a variety of pathological contexts ranging from inflammation to cancer (Nakata et al., 2006; Chen et al., 2008; Malik et al., 2008). Several studies have shown that in human primary leukocytes, Src family members, particularly Lyn and Lck, are required for CXCR4-dependent cell movement and infiltration into various inflamed tissues (Inngjerdingen et al., 2002; Malik et al., 2008). The SDF-1-mediated activation of Lyn kinase in monocytes, modifies integrin activity through inside-out signaling, and transiently destabilizes monocyte/endothelial cell interactions, facilitating full monocyte detachment from endothelium and penetration into inflamed tissue (Nakata et al., 2006; Chen et al., 2008; Malik et al., 2008). Importantly, Lyn is also required for TNF-α and IL-1β production in inflammatory macrophages during stimulation with the CCR5 receptor ligands (Tomkowicz et al., 2006; Cheung et al., 2008, 2009). The other Src family members, Src and Hck, have been shown to play a critical role in IL-6 production in osteoblasts and inflammatory macrophages, respectively (Smolinska et al., 2011; Peruzzi et al., 2012). IL-6 is required for the maintenance and progression of pancreatic cancer precursor lesions, and thus is required for pancreatic cancer growth (Zhang et al., 2013).

In summary, Src family kinases have been demonstrated to be important in the activation of macrophage, dendritic cells, neutrophils and natural killer cells in normal tissues (Ptasznik et al., 1995, 1996; Abram and Lowell, 2008; Malik et al., 2008). It has also been shown to control production of cytokine TNF-alpha stimulated by LPS in normal cells (Orlicek et al., 1999; Sarang et al., 2011; Okenwa et al., 2013). Thus, Src affects both innate and adaptive immune responses in normal cells. Consequently, the elevated and dysregulated Src activity may play a key role in initiation of the invasive cell phenotype both in infiltrating immune cells and precancerous cells. However, its most robust effects are from the production of cytokines and alterations of cell movement/attachment. In fact, the Src family kinase signaling network is the go between that relay crucial cytokine signals from inflammatory cells to cancer cells, and conversely, within the tumor microenvironment (Figure 1). The Src-mediated stimulatory effects on malignant cell proliferation and inhibitory effect on cell death, leads to the accumulation of malignant cells and thus increases the total mass of the tumor. Consequently, this elevates the production of pro-inflammatory cytokines, including chemokines, by the tumor which further leads to the recruitment and activation of additional leukocytes that results in a cycle (as depicted in the Figure 1) leading to cancer development and progression.

## Concluding remarks

The Src kinases-dependent signaling that link immune system with normal tissue plays a vital role in regulating and coordinating immune defense responses. The cross talk between Src kinase pathways in immune cells and Src kinase-mediated pathways in target tissue cells is mediated via cytokine signals elicited by these cells. These Src-dependent signaling pathways, when hyper-activated and dysregulated, can lead to the development of chronic inflammation that predispose to cancer. Src activation both in infiltrating immune cells and cancer precursor lesions is driven by pro-inflammatory cytokines within tumor-promoting microenvironment. This leads to a vicious cycle in which Src activation increases cytokine production that again induces Src activation, leading to invasive inflammatory cell and cancer cell phenotypes. Thus, elucidating the Src-dependent cross talk signaling mechanisms that link inflammatory cells with cancer cells, may facilitate the design of new pharmacological agents for the concurrent treatment of tumor-promoting inflammation and cancer. Pancreatic cancer, because of its robust cytokine mediated interactions between the tumor cells and tumor microenvironment, can be used in designing new agents for the inhibition of the linkage between inflammation and cancer.

### Conflict of interest statement

The authors declare that the research was conducted in the absence of any commercial or financial relationships that could be construed as a potential conflict of interest.

## Abbreviations

LPS: lipopolysaccharides
TNF-α: tumor necrosis factor
IL-1: interleukine 1
IL-6: interleukine 6
MCP-1: monocyte chemoattractant protein-1
MIP-1: macrophage inflammatory protein 1
MIP-2: macrophage inflammatory protein 2
SDF-1: stromal cell-derived factor 1
PI3K: phosphatidylinositol-3 kinase.
